# Engineering CHO cells for improved central carbon and energy metabolism

**DOI:** 10.1186/1753-6561-5-S8-P120

**Published:** 2011-11-22

**Authors:** Camila A Wilkens, Ziomara P Gerdtzen

**Affiliations:** 1 Centre for Biochemical Engineering and Biotechnology, Department of Chemical Engineering and Biotechnology, University of Chile, Santiago, 8370448, Chile; 2 Millennium Institute for Cell Dynamics and Biotechnology: a Centre for Systems Biology, University of Chile, Santiago, 8370448, Chile

## Background

Investigations have shown animal cell cultures’ performance, in terms of cell proliferation and production of recombinant protein, are negatively affected by both lactate's concentration and its specific production rate. In a previous work, we determined that lactate production was caused by pyruvate accumulation due to its high synthesis rate in the glycolitic pathway and limited consumption in the TCA cycle, which leads to lactate production [1]. In this work, we use the ΔL/ΔHexose ratio in order to characterize the cells metabolic state. This ratio describes the lactate production rate *vs.* hexose consumption. Low ΔL/ΔHexose ratios indicate efficient metabolic states where carbons consumed are mainly used to support cell growth, protein synthesis or energy metabolism.

Cell engineering has been previously used to improve cultures’ performance by changing the expression of genes involved in metabolism and apoptosis, focusing on the modification of only one gene at the time. These works showed that after overexpression of genes such as fructose transporter (Slc2a5) and yeast’s pyruvate carboxylase (PYC) cells are able to achieve higher cell densities and lower lactate production than wild-type cells under the same culture conditions [2-4].

In this work we aim at introducing multiple changes in the cells’ genome in order to obtain an engineered cell line with reduced lactate production and enhanced energy metabolism, which is capable of achieving higher cell densities and with a longer lifespan. We propose to control both, carbon uptake and its use by the TCA cycle. Cells were transfected with the fructose transporter gene (Slc2a5) and pyruvate carboxylase gene (PYC). Metabolic flux redistribution was studied through metabolic flux analysis, comparing engineered cells and wild-type under normal culture conditions.

## Materials and methods

**Figure 1:**
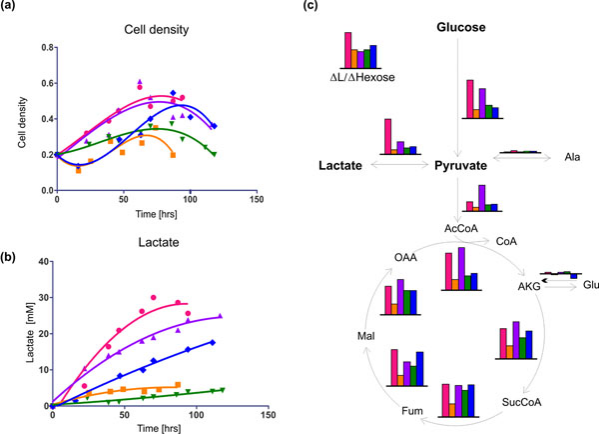
**Experimental and MFA results.** Pink circles: *GC*, orange squares: *FC*, purple upwards triangle: *CHO-PYC* , green downwards triangle: *CHO-Slc2a5* , blue rhombus: *CHO-PYC-Slc2a5*. (a) Cell density, (b) Lactate concentration (c) Comparison of metabolic flux distribution in carbon mmol/10^9.^cells/hr for the different experiments during mid exponential growth. Scale is the same in all graphs.

CHO cells were transfected with the pcDNA3.1(+) zeo-Slc2a5 and/or PCMVSHE-PYC2 + Hygromycine resistance vectors using lipofectamine. After selection, five experiments were designed to study cell proliferation, carbon source consumption, lactate production and metabolic fluxes. CHO cells overexpressing PYC (*CHO-PYC*) were cultured with glucose 17.5 mM and cells transfected with Slc2a5 (*CHO-Slc2a5*) and both PYC and Slc2a5 (*CHO-PYC-Slc2a5*) were grown in media containing fructose 17.5 mM. Two control cultures were performed with wild-type CHO cells in 17.5 mM glucose (*GC*) or fructose (*FC*). Results are shown in Figure 1.

## Results

### Cultures’ performance

As seen in Figure 1.(a) and Table 1 respectively, GC, CHO-PYC and CHO-PYC-Slc2a5 were able to reach higher cell densities and maximum growth rates (µ_max_) than FC and CHO-Slc2a5. Cultures with glucose have almost no lag phase while experiments with media supplemented with fructose have long lag phases, probably due to the slower uptake of fructose, which would delay the exponential growth phase. In addition, engineered cells exhibit an extended lifespan in comparison to wild-type cells.

**Table 1:** Parameters for cell growth and ΔL/ΔHexose

Experiment	µ_max_ [10^-2^ hrs^-1^]	ΔL/ΔHexose
*GC*	1.63	1.7
*FC*	0.86	0.81
*CHO-PYC*	2.1	0.81
*CHO-Slc2a5*	0.65	0.88
*CHO-PYC-Slc2a5*	3.68	1.1

ΔL/ΔHexose values reached by the cultures are given in Table 1. CHO cells grown in high glucose show an inefficient metabolic state where most carbons consumed go towards lactate production. Engineered cells grown in glucose have lower lactate production per carbon consumed than wild-type cells (Figure 1.(b)).

Engineered cells show a better use of glucose, producing less lactate per glucose consumed, as reflected in their lower ΔL/ΔHexose. In addition, CHO-PYC cells are able to produce less lactate and achieve a longer lifespan than wild-type cells. CHO-Slc2a5 cells have higher fructose uptake rates than FC and are able to achieve longer lifespans and higher cell densities.

CHO-PYC-Slc2a5 cells have the highest µ_max_ among all experiments yet they produce more lactate than FC. Most of the lactate is produced in the lag phase. The fact that cells are capable of growing in fructose as well as in glucose and have a better ΔL/ΔHexose than GC, indicates that there is room for further improvement of this system.

### Metabolic flux analysis

Figure 1.(c) shows the flux distribution of the different cultures for central carbon metabolism during mid exponential growth phase. CHO cells grown in fructose have lower amounts of carbon directed towards energy metabolism. Both *CHO-Slc2a5* and *CHO-PYC-Slc2a5* have higher fluxes in glycolysis and TCA cycle than *FC*, consistent with higher cell density. *CHO-PYC* cells consume lower amounts of glucose than *GC*, and most of it is directed towards the TCA cycle. *CHO-Slc2a5* cells consume higher amounts of fructose than *FC* and most of it is directed towards the TCA cycle. *CHO-PYC-Slc2a5* cells show a more active metabolism than *FC*, consuming more fructose, with higher TCA cycle fluxes and lactate production, while reaching higher cell densities than the control.

## Conclusions

It is possible to modify cells for a more efficient metabolism in media supplemented with glucose and fructose using cell engineering. Engineered cells show enhanced viability and more efficient metabolic states under high glucose or fructose concentrations than the controls.
